# Optimizing extended ablation margins in papillary thyroid carcinoma using digital pathology

**DOI:** 10.3389/fendo.2025.1614005

**Published:** 2025-08-15

**Authors:** Han-xiao Zhao, Yun Niu, Zhen-long Zhao, Yu-tong Liu, Ming-an Yu

**Affiliations:** ^1^ China-Japan Friendship Institute of Clinical Medical Sciences, Beijing, China; ^2^ Department of Interventional Medicine, China-Japan Friendship Hospital, Beijing, China; ^3^ Department of Pathology, China-Japan Friendship Hospital, Beijing, China

**Keywords:** papillary thyroid carcinoma, thermal ablation, extended ablation margin, local recurrence, digital pathology

## Abstract

**Background:**

To reduce the risk of local recurrence of papillary thyroid carcinoma (PTC) after thermal ablation, in clinical practice, the ablation area is typically extended 2–3 mm beyond the original tumor margin. However, there is no definitive evidence to support the validity of this extended margin.

**Objective:**

The objective of this study was to measure the distance from the edge of a PTC to the farthest point of tumor infiltration using digital pathology and to analyze the associated risk factors.

**Methods:**

A total of 227 patients who underwent surgical resection for PTC were included in the study. The median age was 45 years. The slides with the maximum tumor diameter were selected as the target slides. Two authors independently assessed the characteristics of the PTCs and measured infiltration distances, with the greatest distance recorded as the infiltration distance. Risk factors associated with infiltration distance were analyzed using the rank-sum test, correlation analysis, and multiple linear regression.

**Results:**

Of the 227 tumors, 23 tumors showed no signs of infiltration; the remaining 204 tumors had infiltration distances ranging from 0.14 to 2.26 mm, with 3 tumors having distances greater than 2 mm (2.17, 2.19 and 2.26 mm). Significant differences in infiltration distances were observed in relation to maximum tumor diameter, lymph node metastases, and tumor growth patterns (TGPs) on the basis of the rank-sum test or correlation analyses. However, multiple linear regression analyses revealed that only TGPs were risk factors for infiltration distance.

**Conclusion:**

To minimize the risk of local recurrence of PTC following thermal ablation, extending the ablation margin by 2.5 mm is recommended, regardless of the tumor stage.

## Introduction

Papillary thyroid carcinoma (PTC) represents 80-90% of differentiated thyroid cancers, making it the predominant histological type (1). Thermal ablation (TA) techniques, such as radiofrequency ablation (RFA) and microwave ablation (MWA), have shown excellent efficacy and a lower incidence of complications in treating T1 PTC (2–4), providing an alternative for patients who decline active surveillance or surgical resection. As a malignant tumor, PTC grows and infiltrates into the surrounding normal thyroid tissue (5). However, the limited resolution of ultrasound (US) makes it difficult to detect small infiltrations around tumors. To reduce the risk of local recurrence of PTC following TA, the ablation area is typically extended by 2–3 mm beyond the original tumor margin on the basis of clinical experience (2, 6). However, there is no definitive evidence to support the validity of this extended ablation margin. In other words, insufficient extended ablation increases the risk of local recurrence, and excessive extended ablation destroys an increased amount of normal thyroid tissue. The aim of this study was to measure the distance from the edge of the PTC to the farthest point of tumor infiltration using digital pathology and analyze risk factors associated with PTC infiltration, providing a scientific basis for optimizing the extended ablation margin to minimize the risk of local recurrence.

## Materials and methods

### Patients

This retrospective study received approval from the Human Ethics Review Committee of China-Japan Friendship Hospital (IRB No. S2019-283-02). The medical data of all consecutive patients with PTC who underwent surgical resection between January and May 2023 at China-Japan Friendship Hospital were reviewed. All patients provided written informed consent for treatment. The need for informed consent for inclusion in the study was waived because all personal details were kept confidential.

### Inclusion and exclusion criteria

**Figure 1:**
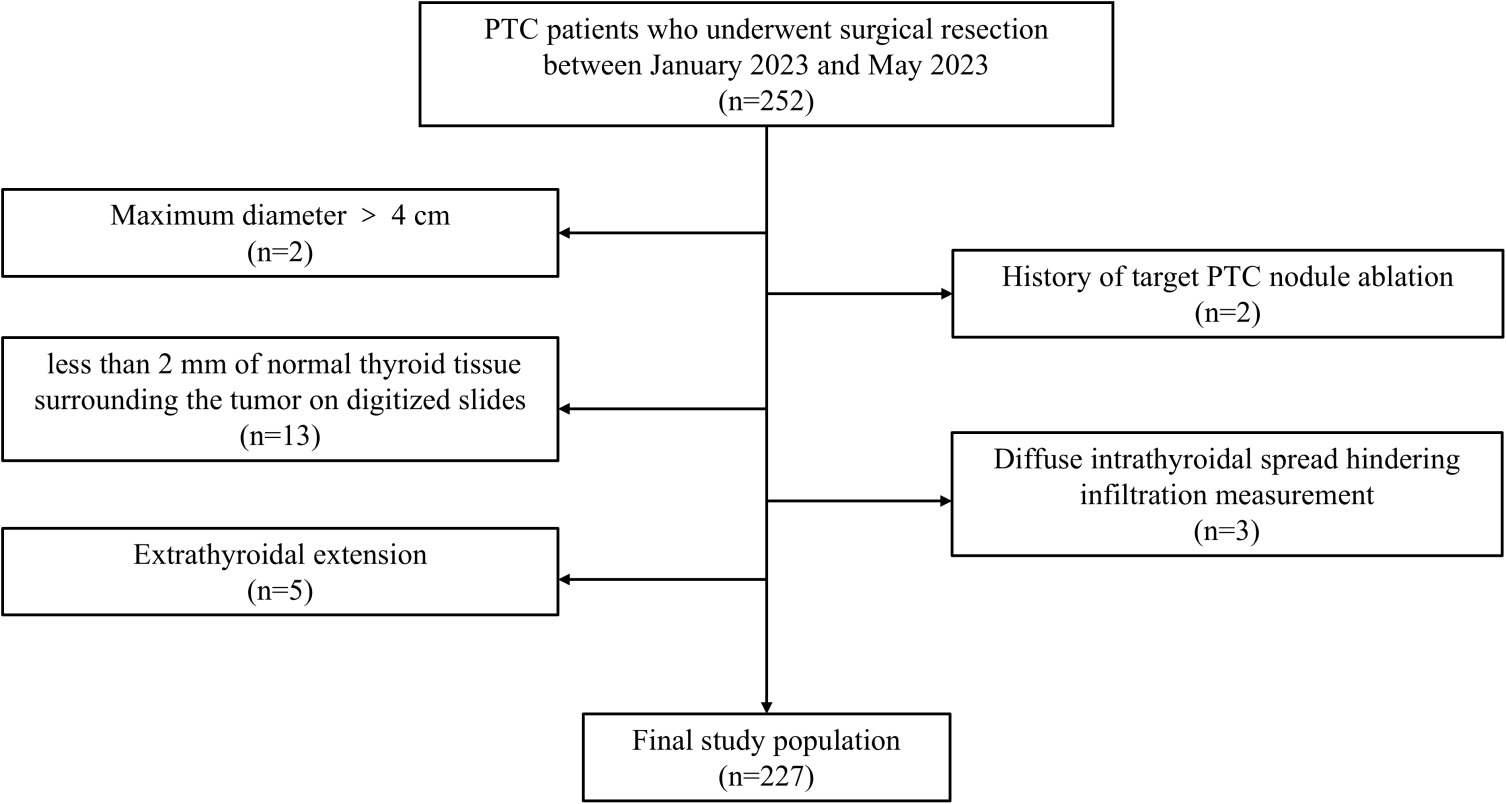
Study flowchart.

**Figure 2:**
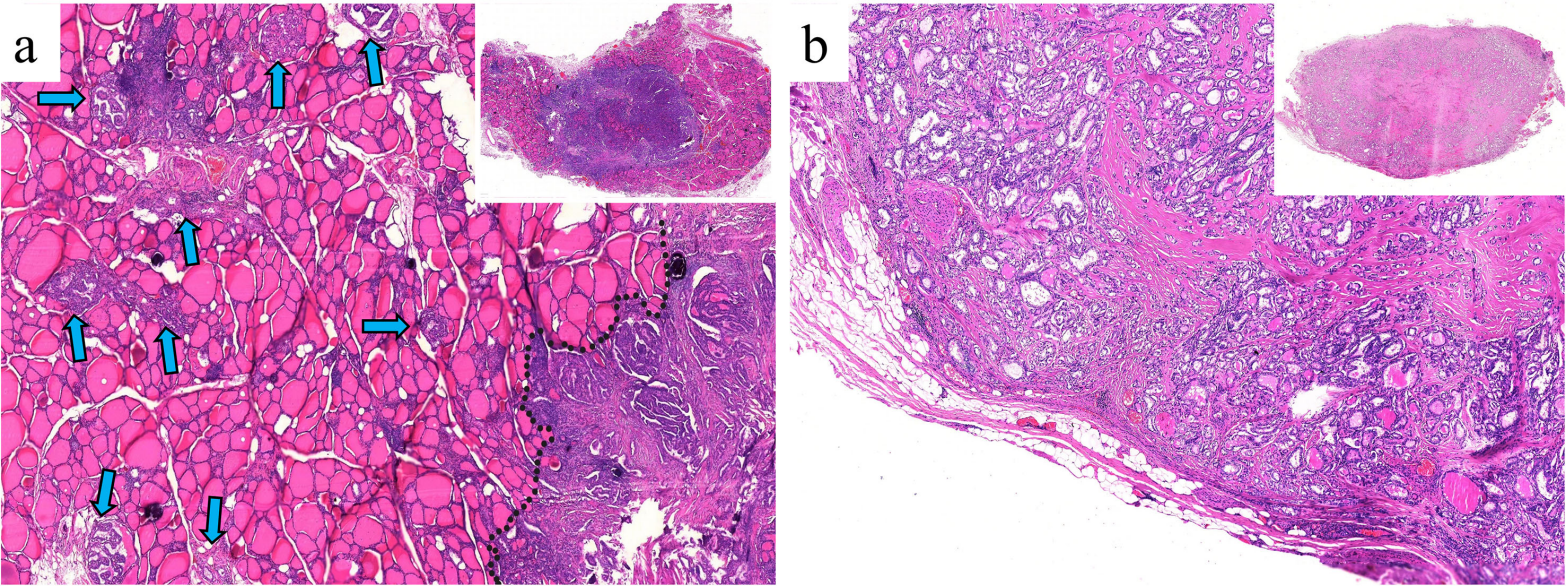
**(a)** Multifocal PTC and lymph node metastasis in a 36-year-old female patient. The largest nodule, measuring 1.2 × 1 × 0.8 cm (black dashed line), is located in the left thyroid lobe. Diffuse intrathyroidal spread is observed (blue arrows), with all spreading foci < 1 mm (H&E, × 100). **(b)** Multifocal PTC without lymph node metastasis in a 30-year-old male patient. The largest nodule, measuring 1.2 × 0.7 × 0.7 cm, is located in the left lobe. The target section shows almost no normal tissue surrounding the nodule (H&E, × 180). These two PTC types were excluded from this study because infiltration distances could not be measured.

The inclusion criteria were as follows: (1) underwent partial or total thyroidectomy; (2) had postoperative pathologically confirmed PTC; (3) had a maximum tumor diameter ≤ 4 cm; and (4) had no extrathyroidal extension. The exclusion criteria were as follows: (1) history of target PTC nodule ablation; (2) diffuse intrathyroidal spread hindering infiltration measurement; and (3) less than 2 mm of normal thyroid tissue surrounding the tumor on digitized slides. The patient selection process is shown in **Figure 1**, and examples of excluded cases are illustrated in **Figure 2**.

### Definitions

**Figure 3:**
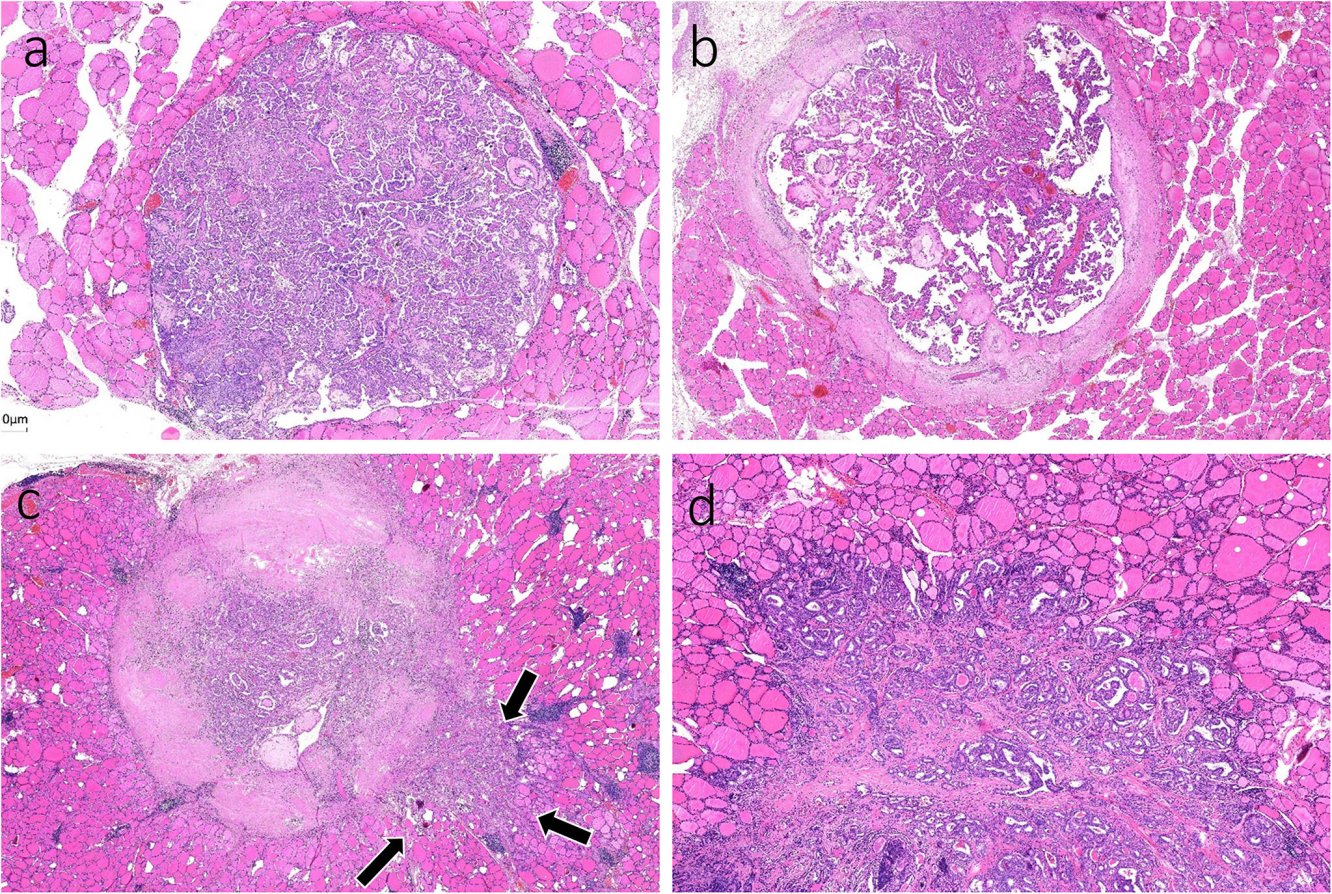
Tumor growth patterns (TGPs). **(a)** TGP I: well-circumscribed growth with no pseudocapsule and obvious infiltration into adjacent normal follicles (H&E, × 80). **(b)** TGP I: well-circumscribed growth completely surrounded by fibrous pseudocapsules (H&E, × 80). **(c)** TGP II: coexistence of encapsulated and infiltrative (black arrows) growth (H&E, × 80). **(d)** TGP III: infiltrative growth with irregular, stellate or spiculated margins (H&E, × 200).

Three kinds of tumor growth patterns (TGPs) were defined: (I) well-circumscribed growth with no obvious infiltration into adjacent normal tissue, regardless of the presence or absence of fibrous pseudocapsules; (II) coexistence of encapsulated (surrounded by fibrous pseudocapsules) and infiltrative growth; and (III) infiltrative growth with irregular, stellate, or spiculated margins (7, 8). (**Figure 3**) On the basis of the 2022 WHO classification, PTC was categorized into classic PTC, infiltrative follicular variant, and other types (9). In this study, the “other types” category comprised only two cases: one columnar cell variant and one hobnail variant. Capsular invasion was defined as neoplastic infiltration of the thyroid fibrous capsule (10). Calcifications included psammoma bodies (spherical calcified foci with concentric laminations) and stromal calcifications (spherical without laminations or irregular with laminations) (11). Thyroid status was categorized as either normal or Hashimoto thyroiditis. The diagnosis of Hashimoto thyroiditis was based on strict criteria: (1) preoperative thyroid peroxidase antibody level > 34 IU/mL and/or antithyroglobulin antibody level > 115 IU/mL; and (2) postoperative histopathology (12).

### Image acquisition for digital pathology

Thyroid gland specimens obtained from unilateral or bilateral lobectomies were analyzed. The head side of each sample was marked by surgeons to guide the preparation of longitudinal pathological sections. Postoperative tissues were fixed with 10% formaldehyde, cut into 4-μm sections and stained with hematoxylin and eosin (H&E). In patients with multifocal PTC, the largest nodule was identified as the dominant nodule. Slides with the maximum diameter from the unifocal nodule or the dominant nodule in multifocal cases were selected as the target slides. If the tumor required dissection due to its larger volume, all the slides with the maximum diameter were designated as target slides. All target slides were scanned and input into whole slide imaging (WSI) at × 40 magnification.

### Digital image analysis

**Figure 4:**
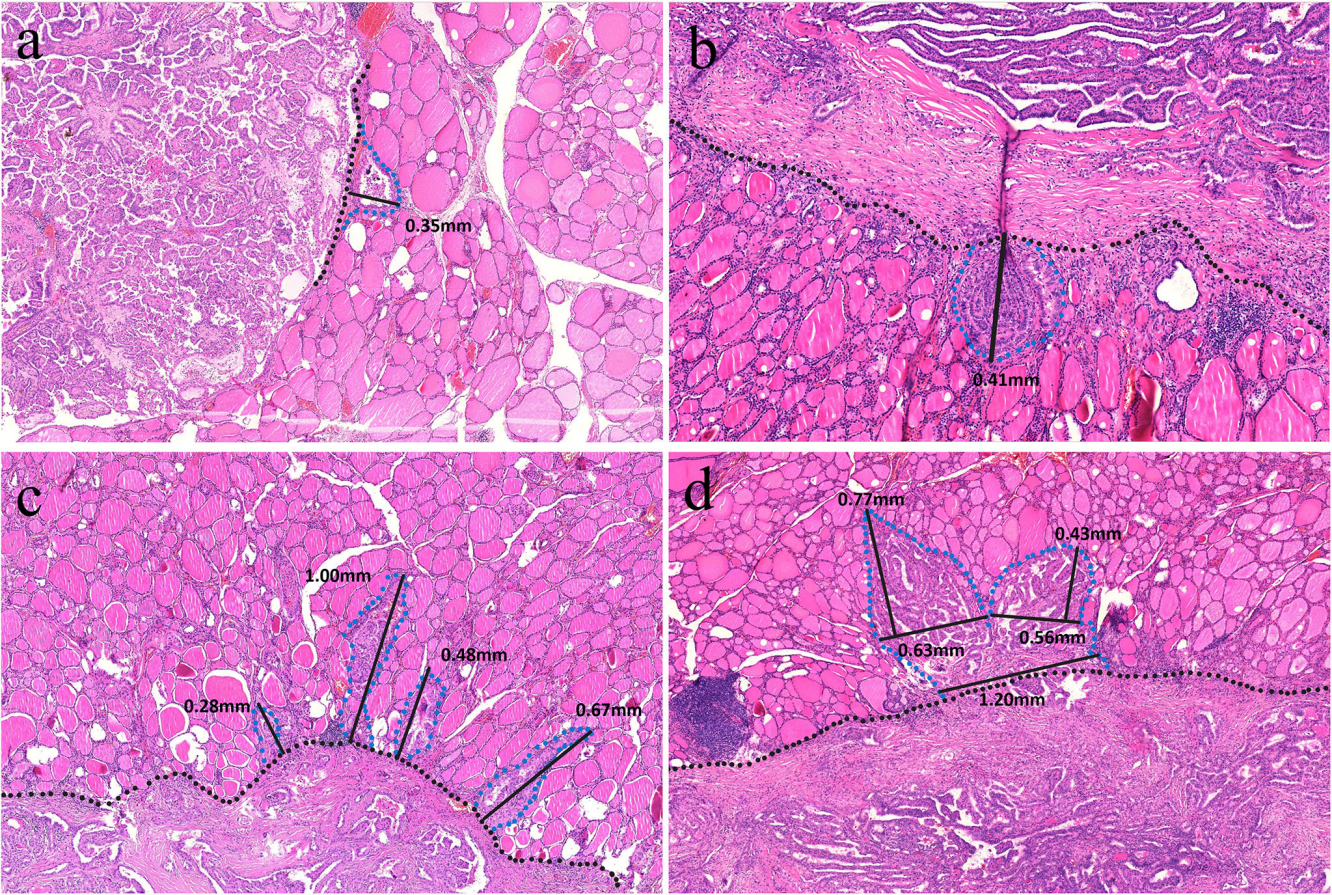
**(a)** A TGP I tumor is well circumscribed without pseudocapsules, and the vertical distance from the farthest point of the tumor to the outer boundary is 0.35 mm (H&E, × 160). **(b)** A TGP I tumor is well circumscribed with fibrous pseudocapsules, and the vertical distance from the farthest point of the tumor to the fibrous pseudocapsule is 0.41 mm (H&E, × 320). **(c)** A TGP III tumor shows infiltrative growth with spiculated margins, and the base width of each spiculated infiltration is < 1 mm. The vertical distance from the farthest point of infiltration to the boundary was measured, and the greatest distance of 1.00 mm was used (H&E, ×200). **(d)** A TGP III tumor shows infiltrative growth with stellate margins, and the base width of stellate infiltration is > 1 mm. A base width of < 1 mm was used as the boundary, and the vertical distance from the farthest point of infiltration to the boundary was measured, with the greatest distance of 0.77 mm (H&E, ×180).

To improve accuracy, two authors (H. X. Zhao and Y. Niu) independently analyzed all digitized slides using ImageJ software. Discrepancies were resolved through joint review of the slide until consensus was achieved. The infiltration distance for TGP I tumors was determined by the vertical distance from the farthest point of tumor infiltration to the outermost boundary or fibrous pseudocapsules. Owing to the limited resolution of ultrasound, the 7.5–15 MHz probe employed in thyroid examinations can generally identify spiculation or lobulation approximately 1 mm near the tumor (13, 14). The aim of this study was to investigate infiltration distances that cannot be visualized using high-resolution ultrasound. Therefore, for TGP III tumors, the base of infiltration was established at a boundary width of 1 mm, measured as the infiltration distance from the farthest point of the tumor cells to this boundary. In TGP II tumors, infiltration distances were assessed by both methods previously described. The greatest measured distance for each tumor was considered the definitive infiltration distance. (**Figure 4**).

### Statistical analysis

Statistical analysis was performed with SPSS (version 26.0). In terms of descriptive statistics, variables that did not follow a normal distribution are described as medians and interquartile ranges, and categorical variables are presented as numbers with percentages. To compare infiltration distances between different groups, rank-sum and grouped rank-sum tests were performed, with Bonferroni correction applied for intragroup comparisons. Rank correlation analysis was used to assess the correlation between the maximum diameter of the PTC and infiltration distance. Risk factor analysis for infiltration distances was conducted using multiple linear regression, with TGP I and classic PTC included as dummy variables. A difference in the data with P < 0.05 was considered to indicate statistical significance.

## Results

### Patient characteristics

A total of 227 patients (63 males and 164 females) who underwent surgical resection for PTC were included in the present study. The median age was 45 years (25–75% IQR 36–56, age range 23–81). A total of 118 patients were in stage T1a, 92 were in stage T1b, and 17 were in stage T2. The median maximum tumor diameter was 1.0 cm (25-75% IQR 0.6-1.5, diameter range 0.3-4.0). LNMs were observed in 119 patients (52.4%), multifocal nodules in 102 (44.9%), capsular invasion in 182 (80.2%), and calcification in 91 patients (40.1%). With respect to TGP classification, 75 patients were classified as TGP I, 61 as TGP II, and 91 as TGP III. (**Table 1**).

**Table 1:** Baseline patient characteristics.

Characteristics	Data
Age	45 (36-56)
Sex	
Female	164 (72.2%)
Male	63 (27.8%)
Tumor stage	
T1a (≤ 1 cm)	118 (52.0%)
T1b (> 1 cm and ≤ 2 cm)	92 (40.5%)
T2 (> 2 cm and ≤ 4 cm)	17 (7.5%)
Maximum tumor diameter, cm	1.0 (0.6-1.5)
Lymph node metastasis	
Present	119 (52.4%)
Absent	108 (47.6%)
Tumor focality	
Unifocal	125 (55.1%)
Multifocal	102 (44.9%)
Tumor variants	
Classic	198 (87.2%)
Infiltrative follicular	27 (11.9%)
Others	2 (0.9%)
Tumor growth patterns (TGPs)	
TGP I	75 (33.0%)
TGP II	61 (26.9%)
TGP III	91 (40.1%)
Capsular invasion	
Present	182 (80.2%)
Absent	45 (19.8%)
Calcification	
Present	91 (40.1%)
Absent	136 (59.9%)
Thyroid function	
Hashimoto thyroiditis	69 (30.4%)
Normal	158 (69.6%)
Infiltration distance, mm	0.51 (0.34-0.78)

Data are presented as the median (25–75% interquartile range) or number of patients (percentages).

### Infiltration distances

Among the 227 tumors, 23 tumors showed no signs of infiltration. The remaining 204 tumors had infiltration distances ranging from 0.14 to 2.26 mm, with 3 tumors having distances > 2 mm, measuring 2.17, 2.19 and 2.26 mm (**Table 2**). The tumor infiltration distances did not follow a normal distribution, with a median of 0.51 mm (25-75% IQR 0.34-0.78; range: 0–2.26 mm). In terms of tumor stage, the median infiltration distance was 0.46 mm for T1a tumors (25-75% IQR 0.34-0.75; range: 0–2 mm), 0.57 mm for T1b tumors (25-75% IQR 0.33-0.86; range: 0-2.26 mm), and 0.41 mm for T2 tumors (25-75% IQR 0.26-0.90; range: 0-1.36 mm). However, there was no significant difference among the three groups (P=0.347).

**Table 2:** Characteristics of three PTC patients with infiltration distances >2 mm.

Case	Age/Sex	Tumor stage	LNM	Tumor focality	Tumor variants	TGPs	Capsular invasion	Calcification	Thyroid function	Infiltration distances
1	62/F	T1b	Absent	Multifocal	Classic	I	Present	Present	HT	2.17 mm
2	70/M	T1b	Absent	Unifocal	Classic	II	Absent	Absent	HT	2.19 mm
3	56/F	T1b	Present	Unifocal	Classic	III	Present	Absent	Normal	2.26 mm

M, Male; F, Female; LNM, Lymph node metastasis; TGP, Tumor growth pattern; HT, Hashimoto thyroiditis.

### Influence factors and multiple linear regression

According to the univariate or correlation analysis, 3 variables (maximum tumor diameter, LNMs and TGP) were significantly associated with infiltration distance (all P < 0.05). As the maximum tumor diameter increased, the infiltration distance also increased (r_s_=0.140, P=0.035). Tumors with LNMs (0.57 mm, 25-75% IQR: 0.38-0.82) presented greater infiltration distances than those without (0.46 mm, 25-75% IQR: 0.28-0.71) (P=0.027). The infiltration distance of tumors in TGP III (0.69 mm, 25-75% IQR: 0.44-0.95) was significantly greater than that in TGP II (0.54 mm, 25-75% IQR: 0.34-0.78, P=0.029) and TGP I (0.35 mm, 25-75% IQR: 0.15-0.58, P < 0.001), with TGP II also having a greater infiltration distance than TGP I (P=0.004). Other variables (age, sex, tumor stage, tumor focality, tumor variants, capsular invasion, calcification, and thyroid function) were not significantly associated with infiltration distance (all P > 0.05) (**Table 3**).

**Table 3:** Univariate or correlation analysis of infiltration distances.

Characteristics	Infiltration distances, mm	z/H/r_s_ value	P
Age	0.51 (0.34-0.78)	-0.098^c^	0.140
Sex		0.410^a^	0.682
Female	0.50 (0.33-0.79)		
Male	0.54 (0.35-0.77)		
Tumor stage		2.116^b^	0.347
T1a (≤ 1 cm)	0.46 (0.34-0.75)		
T1b (> 1 cm and ≤ 2 cm)	0.57 (0.33-0.86)		
T2 (> 2 cm and ≤ 4 cm)	0.41 (0.26-0.90)		
Maximum tumor diameter, cm	0.51 (0.34-0.78)	0.140^c^	0.035*
Lymph node metastasis		2.208^a^	0.027*
Present	0.57 (0.38-0.82)		
Absent	0.46 (0.28-0.71)		
Tumor focality		0.962^a^	0.336
Unifocal	0.51 (0.33-0.84)		
Multifocal	0.52 (0.34-0.77)		
Tumor variants		0.883^b^	0.643
Classic	0.54 (0.34-0.78)		
Infiltrative follicular	0.42 (0.34-0.66)		
Others	0.50/0.77		
Tumor growth patterns (TGPs)		30.50^b^	< 0.001*
TGP I	0.35 (0.15-0.58)		
TGP II	0.54 (0.34-0.78)		
TGP III	0.69 (0.44-0.95)		
Capsular invasion		0.373^a^	0.709
Present	0.51 (0.34-0.78)		
Absent	0.53 (0.28-0.77)		
Calcification		0.751^a^	0.453
Present	0.52 (0.33-0.76)		
Absent	0.50 (0.34-0.82)		
Thyroid function		0.368^a^	0.713
Hashimoto thyroiditis	0.51 (0.35-0.77)		
Normal	0.51 (0.33-0.79)		

The infiltration distances are presented as the median (25–75% interquartile range) or specific figures. The statistical tests used were as follows: ^a^Mann-Whitney U test; ^b^Kruskal-Wallis H test; and ^c^Spearman's rank correlation. *Statistically significant difference (P < 0.05).

**Figure 5:**
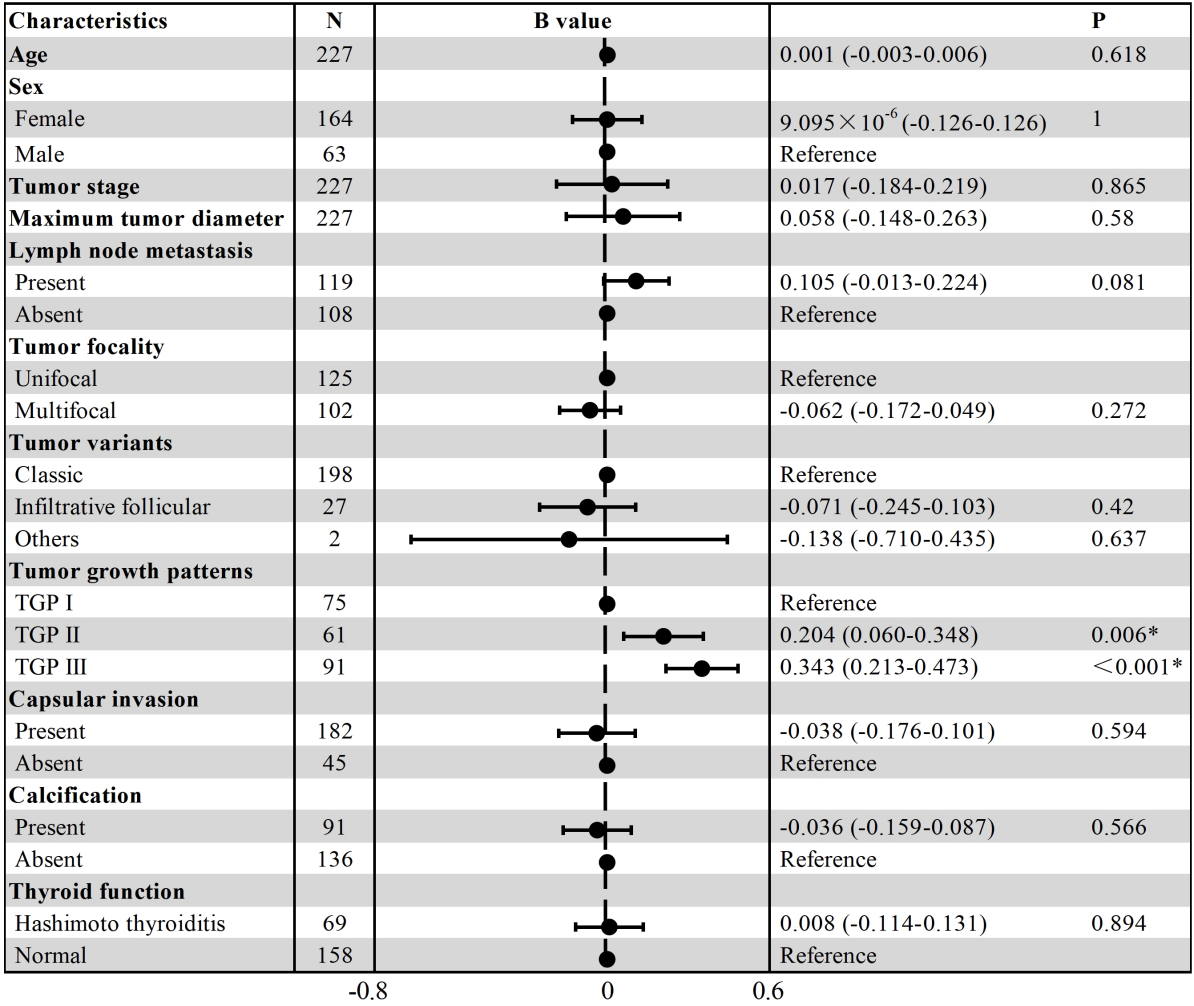
Forest plot of multiple linear regression for infiltration distance.

Multiple linear regression analyses were conducted using the variables mentioned above. The results revealed that only TGPs had a statistically significant effect on infiltration distance. Compared with those with TGP I, tumors with TGP II (B=0.204, t=2.801, P=0.006) and TGP III (B=0.343, t=5.217, P < 0.001) had greater infiltration distances. The other variables did not have statistically significant differences. The results are presented as a forest plot in **Figure 5**.

## Discussion

With advancements in diagnostic technologies such as higher-resolution ultrasound and fine needle aspiration, the detection rate of PTC has significantly increased, contributing to an increase in its incidence (15). While surgical resection remains the traditional treatment for PTC with a favorable prognosis, it can result in postoperative hypothyroidism and the need for lifelong thyroid hormone supplementation (16). Some experts recommend active surveillance for low-risk PTC patients; however, this approach may induce psychological stress and carry the risk of tumor progression and LNMs (17, 18). Thermal ablation has recently emerged as an alternative for patients who refuse surgical resection or active surveillance, demonstrating promising safety and efficacy not only for unifocal stage T1a tumors but also for multifocal or stage T2 tumors (6, 19, 20).

Malignant tumors, including PTC, commonly infiltrate surrounding normal tissues. Owing to the limited resolution of US, minor infiltration by malignant tumors outward is usually undetected. Vasiniotis et al. reported that in malignant liver tumors, biopsy-proven complete tumor ablation with margins of at least 5 mm achieves optimal local tumor control (21). However, owing to the small size of the thyroid, achieving an ablation margin of at least 5 mm is impractical. Therefore, current clinical guidelines typically recommend extending the ablation area by 2–3 mm beyond the PTC margin to minimize local recurrence (2, 6). However, this recommendation lacks robust evidence-based support, and few studies have validated its rationality and effectiveness. The aim of this study is to investigate the specific infiltration distances of PTCs using a digital pathology approach based on WSI scanning and analyze the factors influencing these distances, providing scientific evidence for optimizing the extended ablation margin.

In this study, all included tumors had measured infiltration distances within 2.5 mm. Although Spearman correlation analysis revealed a significant association between the maximum tumor diameter and infiltration distance (P=0.035), no association with tumor stage was found (P= 0.347). Moreover, multiple linear regression did not identify the maximum tumor diameter as a risk factor (P=0.580). Although some studies suggest a potential link between the maximum diameter of the PTC and tumor aggressiveness (capsular invasion, LNMs or extrathyroidal extension) (22–25), other studies indicate that tumor size is not a reliable predictor of aggressiveness, as even small PTCs can exhibit significant aggressiveness (26, 27). In the 2022 WHO Classification of Thyroid Tumors, the term “papillary thyroid microcarcinoma” (maximum diameter < 1 cm) was also removed (28). To date, no research has been published on the relationship between tumor diameter and infiltration distance, particularly when infiltration is undetectable by US. The infiltration distance may reflect tumor aggressiveness to some extent, but according to our results, it does not correlate with tumor diameter in multiple linear regression analysis. The same applies to LNMs. Although the analysis revealed greater tumor infiltration distances in patients with LNMs than in those without LNMs (P=0.027), multiple linear regression indicated that the presence of LNMs was not statistically significant (P=0.081). Therefore, the maximum tumor diameter and LNMs should not be used as references for adjusting the ablation margin.

TGP based on fibrous pseudocapsule integrity was the only factor affecting infiltration distance according to multiple linear regression (P_TGP I vs. TGP II_ = 0.006; P_TGP I vs. TGP III_ < 0.001). The integrity of fibrous pseudocapsules may directly influence the invasive potential of PTCs into surrounding tissues, with the median infiltration distance increasing as fibrous pseudocapsule integrity decreases (TGP I vs. TGP II vs. TGP III: 0.35 vs. 0.54 vs. 0.69 mm). An early study indicated that PTCs with a total capsule were less aggressive, whereas partially encapsulated PTCs showed no difference in aggressiveness (bloodborne metastases or tumor deaths) compared with PTCs lacking capsules (29). Giani et al. reported that classical PTC with an intact capsule is associated with an excellent prognosis; however, once the integrity of the capsule is destroyed, the prognosis significantly worsens, and they recommended redefining encapsulated classical PTC as a low-risk neoplasm (30). In the present study, although the infiltration distance of PTCs in TGP I was shorter than that in other TGP types, an intact fibrous pseudocapsule did not prevent tumor cells from infiltrating surrounding tissues. Among the 75 PTCs in TGP I, only 17 had no infiltration into adjacent thyroid tissues (17/75, 22.67%). The other PTCs showed varying degrees of outward infiltration (0.14-2.17 mm), which suggests that even when the PTC has an intact fibrous pseudocapsule, extended ablation remains necessary.

In this study, the infiltration distances and risk factors were analyzed from a histopathological perspective using digital pathology, providing evidence-based support for extending ablation distances. For patients who are appropriate candidates for thermal ablation therapy, our measurement data suggest that extending the ablation margin to 2.5 mm would be sufficient regardless of tumor stage. However, it should be emphasized that surgical resection remains the guideline-recommended standard treatment, particularly for patients with aggressive variants or LNMs. Preoperative imaging and cytological examination cannot identify these detailed pathological features. Thermal ablation, as a minimally invasive approach, has inherent limitations in identifying aggressive variants and addressing imaging-occult LNMs.

There were several limitations in the present study. First, there were inherent biases related to the single-center nature of the study. Second, the study was limited to slides with the maximum tumor diameter, preventing a comprehensive assessment of PTC infiltration in three-dimensional space. Third, the limited number of patients with other PTC variants (only two cases: one columnar cell variant and one hobnail variant) may have introduced statistical bias in assessing the impact of pathological variants on infiltration distances. Further studies with larger cohorts are needed to evaluate whether aggressive variants require larger ablation margins. Fourth, although patients with diffuse intrathyroidal spread were excluded on the basis of pathological examination, such spread may not be detectable by preoperative ultrasound, potentially leading to inappropriate patient selection for thermal ablation in clinical practice.

In conclusion, on the basis of our findings, extending the ablation margin by 2.5 mm may be appropriate to minimize the risk of local recurrence of PTC following thermal ablation, regardless of tumor stage.

## Data Availability

The original contributions presented in the study are included in the article/supplementary material. Further inquiries can be directed to the corresponding author.
